# Supplementary material for: Patterns of Intron Gain and Loss in Fungi
Source: PLoS Biol. 2004 Nov 30;2(12):e422. doi: 10.1371/journal.pbio.0020422 (PMC532390; doi:10.1371/journal.pbio.0020422)
Supplement: Table S1 — Also available at http://genes.mit.edu/NielsenEtAl/. (4.3 MB ZIP). [file pbio.0020422.st001.zip › NielsenEtAl/html/564.html]

AN1208.1.NCU02966.1.MG05616.1.FG01337.1


```
 CLUSTAL W (1.82) Multiple Sequence Alignments - Introns Inserted


Sequence 1: MG05616.1	510 aa
Sequence 2: FG01337.1	457 aa
Sequence 3: NCU02966.1	504 aa
Sequence 4: AN1208.1	452 aa
Alignment Length: 532 aa
Number Identitical Residues: 267 aa
Alignment Score (without introns) 12129


MG05616.1 	MAQDSVEGPAD------AALQALIIKNAKQTRDIFT--DITS-------RKRIKLVDPAT
NCU02966.1	MAAEVANSAANGSGPDTNPLSTMIRENARKTKLIYAATATTTDASGTSGRKRLKLDPGLA
FG01337.1 	METPEVLQDA-------LQLGALATQNATVSRTLYSHAQATS-------TKRQKLYDAS-
AN1208.1  	MDVIPSTTP-----------GEAVRISAKRTAELFG-------------PEYLMVTPSAS
          	*                         .*  :  ::               :   :    :

MG05616.1 	PTDGDLDRDTLALRFNSEYADLRELPPALAARQVAAAGGRKKPGARKAAAAGPPADTPEE
NCU02966.1	SEDPDITKTGLSLRLHAEYSDVQTLPEAIASKLPAGGPRKK-----KAKPAIEEAPSKSE
FG01337.1 	------EDPIMKRRFRNEYADVETLPPSITAKLPTKQPGKK----TKAGALVRPA-----
AN1208.1  	N-----GSIGVSYRRKTEYEHVKELPPALAAKQAQAAAARS----KRPKISSRSEGSGSG
          	          :  * . ** .:. ** :::::       :.     :.        : . 

MG05616.1 	EKSRKLIEGIPTRG-ANPRS------QNGTGAAQSSALVLKGRGTGTEGQGGQEQRQQQK
NCU02966.1	EHARKLIEGIPPSKSAAANSNALVLSRSKPGGAGASSTRPNAQ------------RNEPQ
FG01337.1 	---MKLLEGAPGSGKASASA------ARNEGTPQNMSLTTRG-----------------T
AN1208.1  	DKSGASTALVRRGGRASGAA----------GDDKPTSLIQRPS-----------------
          	. :            *   :          *     :   .                   

MG05616.1 	QNDRGPPVIGQQLTLARAQELNMQLKPEWHRPWKLMRVISGHLGWVRALAVEPDNKWFAS
NCU02966.1	TN-----------SLARRSDVLVQPRPDWHPPWKLLKVLSGHLGWVRALAVEPDNKWFAS
FG01337.1 	QN-------------------TMQQKPEWHAPWKLMRVISGHLGWVRSLAVEPGNKWFAS
AN1208.1  	--------------------ATRQQPPEWHAPWKLMRVISGHLGWVRSLAVEPNNEWFAS
          	                       *  *:** ****::*:********:*****.*:****

MG05616.1 	GAGDRTIKIWDLASGRLRLTLTGHISTVRGLAVSPRHPYLFSCAEDKMVKCWDLETNKVI
NCU02966.1	GAGDRTIKIWDLASGALKLTLTGHISTVRGLAVSPRHPYLFSCGEDKMVKCWDLETNKVI
FG01337.1 	GAGDRTIKIWDLASGSLKLTLTGHISTVRGLAVSPRHPYLFSCGEDKMVKCWDLETNKVI
AN1208.1  	GAGDRTIKIWNLATGALRLTLTGHISTVRGLAVSPRHPYLFSCGEDKMVKCWDLETNKVI
          	**********:**:* *:*************************.****************

MG05616.1 	RHYHGHLSGVYTLALHPTLDVLVTGGRDGVARVWDMRTRSNIHVLSGHTGTVSELVCQEA
NCU02966.1	RHYHGHLSGVYTLALHPTLDVLVTGGRDGVARVWDMRTRSNIHVLSGHTGTVADLVCQEA
FG01337.1 	RHYHGHLSGVYTLALHPTLDVLVTGGRDGVARVWDMRTRSNIHVLSGHTQTVSDLVCQEA
AN1208.1  	RHYHGHLSGVYTLALHPRLDLLVTGGRDGVARVWDMRTRSNIHVLSGHTGTVADVQCQEA
          	***************** **:**************************** **::: ****

MG05616.1 	DPQVITGSLDSTVRMWDLAAGKTMGVLTHHKKGVRALATHPTEFTFASGSAGSIKQWKCP
NCU02966.1	DPQVITASLDSTVRMWDLAAGKTMGVLTHHKKGVRALTTHPTEFTFATGSTGSIKQWKCP
FG01337.1 	DPQVITGSLDSTVRLWDLAAGKTMGVLTHHKKGVRALATHPSEFTFASGSTGSVKQWKCP
AN1208.1  	DPQVITGSLDATVRLWDLAAGKTMGVLTHHKKGIRSLATHPREFTFASASTGSIKQWKCP
          	******.***:***:******************:*:*:*** *****:.*:**:******

MG05616.1 	EGAFMQNFEGHNSIINTLSVNDQNVFFSGA~DNGSMSFWDWKTGHRFQSLDTTAQPGSLQ
NCU02966.1	EGAFMQNFEGHNAIINTLSVNDQNVMFSGG~DNGSMSFWDWKSGHRFQALDTIAQPGSLD
FG01337.1 	EGAFMQNFEGHNSIINTMSVNEQNVFFTGG1DNGSMSFWDWKTGHRFQSLDTTAQPGSLD
AN1208.1  	GGEFMQNFEGHNAIINTLSVNEDNVLFSGG~DNGSMSFWDWKTGYRYQTIDTTAQPGSLE
          	 * *********:****:***::**:*:*. ***********:*:*:*::** ******:

MG05616.1 	AESGIFTSTFDQSGSRLICGEADKTI~KIWKEDMEATPETHPLDWKPTLGRRKF
NCU02966.1	AESGIMSSTFDKSGARLICGEADKTI1KIWGEDLSATPESHPLEWKPTLAKRKF
FG01337.1 	AEAGIMSSTFDRSGLRLICGEADKTI1KVWKQDETATEESHPLEWKPTLARRKF
AN1208.1  	AEAGIMTSTFDRTGLRLITGEADKTI~KVWKQDDQATPETHPVTWAPTLGRQRY
          	**:**::****::* *** ******* *:* :*  ** *:**: * ***.::::
```
